# Well-deserved Recognition

**Published:** 1897-09

**Authors:** 


### WELL-DESERVED RECOGNITION.

It is gratifying to read in many foreign journals and publications reference to and acceptance of the results of original investigation as to the character and causes of many of the infectious and contagious diseases of live-stock made by such zealous and able workers as Drs. Theobald Smith and Cooper Curtice, of this country, and we are surely all very greatly indebted to these contributors in so great a measure to American veterinary literature.

Our Bureau of Animal Industry has been especially fortunate in having among its corps of workers Drs. Salmon, Smith, Curtice, Moore, Hassall, Kilborne, and others.

